# eHealth Literacy Assessment Instruments: Scoping Review

**DOI:** 10.2196/66965

**Published:** 2025-08-20

**Authors:** Chen Wang, Luoyuan Chang, Xindou Chen, Jingqi Kong, Huiying Qi

**Affiliations:** 1 Department of Health Informatics and Management, School of Health Humanities, Peking University, 38 Xueyuan Road, Haidian District, Beijing, 100191, China, 86 1082802419; 2 School of Health Humanities, Peking University, Beijing, China; 3 School of Public Health, Peking University, Beijing, China

**Keywords:** eHealth literacy, systematic review, assessment, assessment instruments, literacy, digital literacy, eHealth, behavior, behavior interventions

## Abstract

**Background:**

eHealth literacy is a necessary competency for individuals to achieve health self-management in the digital age, and the evaluation of eHealth literacy is an important foundation for clarifying individual eHealth literacy levels and implementing eHealth behavior interventions.

**Objective:**

This study reviews the research progress of eHealth literacy assessment instruments to offer suggestions for further development and improvement as well as to provide a reference to eHealth intervention.

**Methods:**

We reviewed papers on Web of Science, Scopus, PubMed, and EBSCO in English between 2006 and 2024 and included studies involving the development of eHealth literacy assessment instruments, which must be published in peer-reviewed journals. An analysis in terms of the development process, instrument characteristics, and assessment themes was conducted to reveal the content, features, and application of currently available eHealth literacy assessment instruments.

**Results:**

Searches yielded 2972 studies, of which 13 studies were included in the final analysis. The analysis of the 13 studies indicated that the development of instruments is improving constantly, as the concept of eHealth literacy evolves. In total, 9 of the 13 tools are subjective assessments, with eHealth Literacy Scale being the most widely used. In contrast, the remaining 4 comprehensive assessment tools incorporate objective evaluation criteria. The 13 instruments’ reliability ranged from 0.52 to 0.976. Validity was reported for 12 tools (excluding eHealth Literacy Scale), covering 5 types: content validity, structural validity, discriminant validity, external validity, and convergent validity. Regarding assessment themes, skill factors are involved in many instruments, but psychology factors and information factors are less concerned.

**Conclusions:**

The evaluation of the characteristics of existing eHealth literacy assessment tools in this paper can provide a reference for the selection of assessment tools. Overall, subjective and comprehensive assessment tools for eHealth literacy have their own advantages and disadvantages. Subjective assessment tools have a friendly evaluation method, but their test validity is relatively low. There is a risk of time-consuming and low recognition for comprehensive evaluation tools. Future research should be based on the deepening of eHealth literacy connotation, further verifying the effectiveness of existing eHealth literacy assessment tools and adding objective evaluation dimensions.

## Introduction

### Background

In 2005, the World Health Organization defined eHealth as the dissemination of health resources and health care information through electronic means, enabling health care professionals and users to disseminate and access health information [1]. Based on the concept of electronic health, eHealth literacy was first proposed by Norman and Skinner [2], which refers to the ability to search, understand, and assess health information from electronic sources, as well as to deal with and apply the health information obtained, and eventually to solve health problems [2]. To date, this concept is most widely cited. However, with further research in the field and the increasing availability of electronic media, researchers’ perceptions of the connotation of eHealth literacy have changed. Unlike the concept of Norman and Skinner [2], which emphasizes individual capability to apply health information through electronic methods, subsequent studies on the concept of eHealth literacy are weighed on the interaction between the individual and technology and the individual and health technology service provider [3-7].

Entering the mobile age, electronic equipment gets broad applications, and various electronic health programs keep coming out, which enhance the accessibility of medical resources and participation of individuals in medical decisions, having a positive impact on mass health [1,8,9]. On the other hand, the diversity and varying quality of eHealth resources pose challenges for people with limited health literacy. They struggle to distinguish legitimate health services from fraudulent ones and identify credible information, resulting in low public acceptance and inefficient use of eHealth solutions. As a necessary skill in the field of eHealth, eHealth literacy is the foundation to maximize eHealth effectiveness [2]. The improvement of eHealth literacy is beneficial to the reasonable use of eHealth information resources to promote good health behaviors of the public, which is of great practical significance for promoting the health self-management ability of individuals in the information age.

To study the current situation of individuals’ eHealth literacy levels and design a corresponding intervention to improve their eHealth literacy, a systematically conducted eHealth literacy assessment is the foundation and prerequisite. Meanwhile, appropriate eHealth literacy assessment instruments can not only measure individuals’ capacity to use the eHealth tools and their benefit from these tools [10] but also recognize the special population disabled to effectively use eHealth services and experiencing “the digital divide” [11]. Up to now, the research on eHealth literacy assessment has yielded a series of research outcomes [12-15], whereas a discrepancy exists in the aspects of use scenario, applicable population, assessment themes, and dimensions of the relevant assessment models and tools. Therefore, it is necessary to sort out and analyze systematically eHealth literacy assessment instruments.

### Objective

The aim of this scoping review is to comprehensively review and evaluate the characteristics, effectiveness, and limitations of existing eHealth literacy assessment instruments, providing reference and suggestions for researchers and clinicians in selecting or developing assessment instruments in the future.

## Methods

### Literature Searches

This scoping review was conducted in accordance with the PRISMA (Preferred Reporting Items for Systematic Reviews and Meta-Analyses) statement [11]. A completed PRISMA-ScR (Preferred Reporting Items for Systematic Reviews and Meta-Analyses extension for Scoping Reviews) checklist is included in Checklist 1. Based on the research theme and considering the number of literature collections, update speed, and availability of papers, this study ultimately selected 3 databases (Web of Science, Scopus, and PubMed) and EBSCO; with regard to search strategy, the search string was (“ehealth literacy” OR “E-health literacy” OR “electronic health literacy”) AND (Assessment* OR Measure* OR Tool* OR Test* OR Instrument* OR Questionnaire* OR Psychometric* OR Screen* OR Survey*) and selected “article” as the literature category, and finally, 2972 records were obtained (searched as of June 1, 2024). The search strategies and key terms for all databases can be found in Multimedia Appendix 1.

### Inclusion and Exclusion Criteria

We included studies involving the development of eHealth literacy assessment instruments, which must be published in peer-reviewed journals and written in English. If a study revolved around the theme of eHealth literacy assessment instruments, but the main purpose is not to develop a new eHealth literacy assessment instrument, then the paper was not included. In addition, if a study developed a new assessment instrument but only targeted a specific population, such as older people, the paper was also excluded. Review, commentary, or opinion papers were excluded.

### Study Selection

All records were exported to EndNote (version X9.1; Clarivate Analytics), and duplicate records were identified and deleted. Two reviewers (CW and XC) independently evaluated the literature search results and corresponding full texts based on inclusion and exclusion criteria. If there is a difference between their selection, a discussion and consultation with the third reviewer (LC) was carried out to reach a consensus. Risk of bias was not formally assessed using a specific tool; however, the inclusion criteria were designed to minimize bias by including only original studies with clearly reported methods and outcomes.

### Data Extraction

The review authors (CW and LC) independently extracted data on development time and country, number of dimensions, number of items, scale rating level, reliability and validity testing, dimensions of assessment, and number of corresponding items. Differences in data extraction were resolved through consensus or reference to another author.

### Data Synthesis

We synthesized the collated data by using descriptive statistics. We used Microsoft Excel to analyze the data.

## Results

### Overview

**Figure 1.**
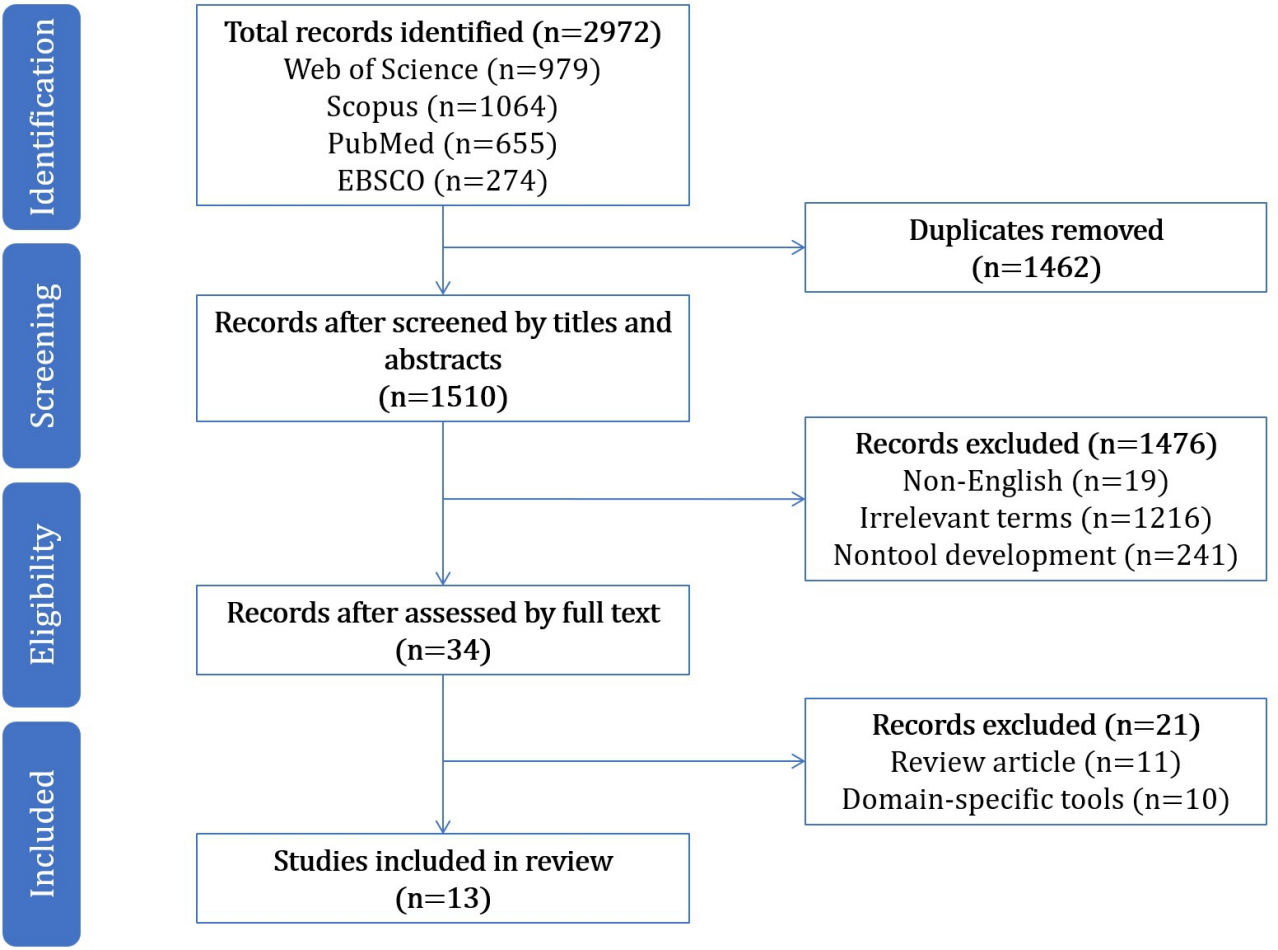
Paper search and screening process.

The database search initially identified 2972 records (979, 1064, 655, and 274 in Web of Science, Scopus, PubMed, and EBSCO, respectively), and 1510 (50.8%) records were screened after removing duplicates (Figure 1). After screening titles and abstracts, 1476 (49.7%) publications were excluded. Of the total of 34 (1.1%) full-text papers screened, 13 (0.4%) papers were included in this review.

### Assessment Instruments’ Basic Information and Development Process

The collected 13 eHealth literacy assessment instruments’ basic information is shown in Table 1, which were developed from 2006 to 2022. The United States accounts for the most in the number of instruments with 4, followed by Denmark and China, both with 2. Based on the development of eHealth literacy concept, related technological environment, and changes in the quantity and quality of assessment tools, we divided the development of eHealth literacy assessment instruments into 2 distinct stages: the primary stage (2006) and the growth stage (2014‐2022).

**Table 1.** Basic information of eHealth literacy assessment instruments.

No	Development time	Abbreviation of instruments	Full name of instruments	Authors	Development country
1	2006	eHEALS	The eHealth Literacy Scale	Norman and Skinner [2]	Canada
2	2006	RRSA	Research Readiness Self-Assessment	Ivanitskaya et al [16]	United States
3	2014	PRE-HIT	The Patient Readiness to Engage in Health Internet Technology instrument	Koopman et al [17]	United States
4	2016	e-HLS	Electronic Health Literacy Scale	Seçkin et al [18]	United States
5	2017	eHEALS-E	Extended eHealth Literacy Scale	Petrič et al [19]	Slovenia
6	2017	DHLI	The Digital Health Literacy Instrument	van der Vaart and Drossaert [20]	Netherlands
7	2018	eHLA	eHealth Literacy Assessment toolkit	Karnoe et al [21]	Denmark
8	2018	eHLQ	eHealth Literacy Questionnaire	Kayser et al [22]	Denmark
9	2019	TeHLI	The Transactional eHealth Literacy Instrument	Paige et al [23]	United States
10	2020	DHLA	Digital health literacy assessment	Liu et al [24]	China (Taiwan)
11	2021	eHLS-Web3.0	eHealth Literacy Scale-Web3.0	Liu et al [25]	China (Hong Kong)
12	2022	DHTL-AQ	Digital Health Technology Literacy Assessment Questionnaire	Yoon et al [26]	Korea
13	2022	DHLC	Digital health literacy competencies	Rachmani et al [27]	Indonesia

In the primary stage, eHealth literacy, as a new concept, was in the early stages of comprehension and investigation, and the technological environment such as the internet was not yet mature, so the development of tools at this stage was relatively slow. Canadian and American researchers took the lead in developing eHealth literacy assessment instruments, among which eHealth Literacy Scale (eHEALS) was the first self-assessment instrument that measured eHealth literacy [2], while Research Readiness Self-Assessment (RRSA) [16] put emphasis on appraising eHealth literacy through actual operation in order to avoid the discrepancy between self-assessment and practical level.

With the rapid development of internet technology and the widespread popularity of mobile devices, as well as the further study of scholars in the field of eHealth literacy, the concept of eHealth literacy is continuously expanding, and the development of assessment tools stepped into the growth stage. At this stage, 11 scales were invented by many countries, with the United States in a leading role. Part of these scales was based on existing tools, while others are novel assessment tools on the basis of the concept and framework of eHealth literacy proposed by scholars. Stemming from eHEALS [2], 7 eHealth literacy assessment instruments were derived, including Patient Readiness to Engage in Health Internet Technology (PRE-HIT) [17], Electronic Health Literacy Scale (e-HLS) [18], Extended eHealth Literacy Scale (eHEALS-E) [19], Digital Health Literacy Instrument (DHLI) [20], eHealth Literacy Assessment (eHLA) [21], digital health literacy assessment (DHLA) [24], and eHealth Literacy Scale-Web3.0 (eHLS-Web3.0) [25], and 4 other instruments were constructed depending on newly proposed eHealth literacy framework or model, including eHealth Literacy Questionnaire (eHLQ) [22], Transactional eHealth Literacy Instrument (TeHLI) [23], Digital Health Technology Literacy Assessment Questionnaire (DHTL-AQ) [26], and digital health literacy competencies (DHLC) [27].

### Dimension Setting and Reliability and Validity Examination of Assessment Instruments

#### Overview

The number of dimensions, number of items, scale rating level, and reliability and validity test results of the 13 collected instruments are presented in Table 2.

**Table 2.** Characteristics of eHealth literacy assessment instruments.

	Abbreviation of instruments	Dimensions, n	Items, n^a^	Scale rating level	Reliability, α	Test-retest reliability (interval)	Validity
1	eHEALS^b^ [2]	6	8	5	.88	0.46‐0.68 (4 time points over 6 months)	—^c^
2	RRSA^d^ [16]	3	1+56	6	.78	—	Content validity
3	PRE-HIT^e^ [17]	8	28	4	.60‐.85^f^	—	Structural validity
4	e-HLS^g^ [18]	3	19	5	.93	—	Structural validity
5	eHEALS-E^h^ [19]	6	20	5	.52‐.81^f^	0.77 (2 weeks)	Content validity, discriminant validity
6	DHLI^i^ [20]	7	21+7	4	.87	—	Content validity, structural validity
7	eHLA^j^ [21]	7	28+16	4	.59‐.94^f^	—	Content validity
8	eHLQ^k^ [22]	7	35	4	.77‐.86^f^	—	Structural validity, discriminant validity
9	TeHLI^l^ [23]	4	18	5	.87‐.92^f^	—	External validity
10	DHLA^m^ [24]	3	10+5	5	.87	—	Content validity, structural validity, convergent validity
11	eHLS-Web3.0^n^ [25]	3	24	5	.976	0.858 (1.5 months)	Content validity, structural validity
12	DHTL-AQ ^o^[26]	4	34	4	.95	—	Content validity, structural validity
13	DHLC^p^ [27]	9	26	5	.97	—	Content validity

a The item number column that contains + is the expression form of the number of self-assessment items + the number of actual operation items.

b eHEALS: eHealth Literacy Scale.

c Not available.

d RRSA: Research Readiness Self-Assessment.

e PRE-HIT: Patient Readiness to Engage in Health Internet Technology.

f α range.

g e-HLS: Electronic Health Literacy Scale.

h eHEALS-E: Extended eHealth Literacy Scale.

i DHLI: Digital Health Literacy Instrument.

j eHLA: eHealth Literacy Assessment.

k eHLQ: eHealth Literacy Questionnaire.

l TeHLI: Transactional eHealth Literacy Instrument.

m DHLA: digital health literacy assessment.

n eHLS-Web3.0: eHealth Literacy Scale-Web3.0.

o DHTL-AQ: Digital Health Technology Literacy Assessment Questionnaire.

p DHLC: digital health literacy competency.

In total, 13 instruments contain different numbers of measurement dimensions, one example of which is e-HLS, which includes 3 dimensions: communication, trust, and action. There are 4 instruments with the least dimensions, RRSA [16], e-HLS [18], DHLA [24], and eHLS-Web3.0 [25], of which each contains 3 dimensions. DHLC [27] has 9 dimensions, which is the most among the instruments.

The self-assessment section of the 13 instruments contains varying numbers of items, with an example item in eHEALS being: I have the skills I need to evaluate the health resources I find on the internet. Each item is set to be rated between 4 and 6. The 5-level scale is the most, including 7 instruments, followed by the 4-level scale with 5 instruments, while only 1 instrument is a 6-level scale.

As for reliability, the 13 instruments’ reliability ranged from 0.52 to 0.976, reflecting differences in the reliability of different tools. Among them, 3 tools conducted test-retest reliability assessment. As the minimum acceptable reliability level is usually 0.70, this indicates that some tools have poor reliability. eHLS-Web3.0 [25] has the highest reliability among all tools. In addition, 8 of the 13 instruments reported overall reliability, while the reliability of the 5 instruments, PRE-HIT [17], eHEALS-E [19], eHLA [21], eHLQ [22], and TeHLI [23], was an interval composed of the reliability of each subtool.

Regarding validity, apart from eHEALS [2], all other assessment instruments reported validity. DHLA [24] incorporated the most kind of validity tests, totaling 3 kinds. The other 12 instruments’ validity reports involved 5 kinds, including content validity, structural validity, discriminant validity, external validity, and convergent validity. Among them, only 2 tools underwent discriminant validity testing; external validity and convergent validity appear separately in 1 tool. The number of documents related to content validity is the highest, with a total of 8, but content validity is a very weak criterion for establishing scale validity. The number of documents involving structural validity ranks second, with a total of 7, while structural validity is more stringent.

In total, 13 instruments show diversity in item form, covering a self-assessment scale and a comprehensive assessment system containing direct measurement. According to the form of assessment items, the instruments can be categorized into subjective assessment instruments and comprehensive assessment instruments.

#### Subjective Assessment Instruments

eHealth literacy subjective assessment instruments generally take the form of self-report, with the help of a scale to obtain respondents’ self-appraisal in the aspects of their own cognition, attitude, behavior, skill level, etc. This review collated 9 subjective assessment instruments, encompassing eHEALS [2], PRE-HIT [17], e-HLS [18], eHEALS-E [19], eHLQ [22], TeHLI [23], eHLS-Web3.0 [25], DHTL-AQ [26], and DHLC [27]. The core skill assessment dimensions of each instrument are shown in Table 3.

**Table 3.** The core skill assessment dimensions of subjective assessment instruments.

Abbreviation of instruments and dimensions of assessment		Corresponding items, n
eHEALS^a^ [2]		
	Traditional literacy	8
	Health literacy	8
	Information literacy	8
	Scientific literacy	8
	Media literacy	8
	Computer literacy	8
PRE-HIT^b^ [17]		
	Health information need	5
	Computer or internet experience	4
	Computer anxiety	4
	Preferred mode of interaction	5
	Relationship with doctor	3
	Cell phone expertise	2
	Internet privacy	2
	No news is good news	3
e-HLS^c^ [18]		
	Communication	2
	Trust	4
	Action	13
eHEALS-E^d^ [19]		
	Awareness of sources	3
	Recognizing quality and meaning	3
	Understanding information	4
	Perceived efficiency	4
	Validating information	3
	Being smart on the net	3
eHLQ^e^ [22]		
	Using technology to process health information	5
	Understanding of health concepts and language	5
	Ability to actively engage with digital services	5
	Feel safe and in control	5
	Motivated to engage with digital services	5
	Access to digital services that work	6
	Digital services that suit individual needs	4
TeHLI^f^ [23]		
	Functional	4
	Communicative	5
	Critical	5
	Translational	4
eHLS-Web3.0^g^ [25]		
	Acquisition	8
	Verification	6
	Application	10
DHTL-AQ^h^ [26]		
	Information and communications technology terms	11
	Information and communications technology icons	9
	Use of an app	9
	Evaluating reliability and relevance of health information	5
DHLC^i^ [27]		
	Information and data literacy	1
	Communication and collaboration	6
	Digital content creation	1
	Safety	5
	Problem-solving	5
	Health information access	2
	Health information management	2
	Health information integration	2
	Health information evaluation	2

a eHEALS: eHealth Literacy Scale.

b PRE-HIT: Patient Readiness to Engage in Health Internet Technology.

c e-HLS: Electronic Health Literacy Scale.

d eHEALS-E: Extended eHealth Literacy Scale.

e eHLQ: eHealth Literacy Questionnaire.

f TeHLI: Transactional eHealth Literacy Instrument.

g eHLS-Web3.0: eHealth Literacy Scale-Web3.0.

h DHTL-AQ: Digital Health Technology Literacy Assessment Questionnaire.

i DHLC: digital health literacy competency.

Norman and Skinner [2] developed the eHEALS based on the Lily model [28], which is the first self-assessment tool surveying eHealth literacy, measuring 6 core-related skills. In total, 8 items are contained in this scale, each of which is scored by the Likert 5-level scoring method. One example item is: I know how to find helpful health resources on the internet. The higher the score, the higher the level of self-perceived eHealth literacy. Because eHEALS can measure eHealth literacy through a brief and relatively simple scale, it has been translated into almost 20 languages, such as Dutch [29], Japanese [30], German [31], Portuguese [32], Spanish [33], Turkish [34], Italian [35], Korean [36], Hungarian [37], Serbian [38], Polish [39], Chinese [40], Greek [41], Norwegian [42], Amharic [43], Swedish [44], Arabic [45], Indonesian [46], and so on, as the most widely used eHealth literacy assessment instrument presently.

Koopman et al [17] developed PRE-HIT based on eHEALS from the perspective of the need and motivation of chronic patients by conducting focused interviews on their experience of using digital health resources. This instrument consists of 8 dimensions, and each dimension contains a different number of items, totaling 28 items, with an example item: If I went on the internet, I would use it to look up things so that I wouldn’t worry about them anymore. At present, this instrument is not yet extensively applied.

Seçkin et al [18] believed that existing eHealth literacy assessment instruments are not able to reveal critical aspects of eHealth literacy, such as appraisal, trust, and the communicative aspects of it as a digital process. In 2016, they conducted a comprehensive literature review to identify key skills relevant to eHealth literacy and developed e-HLS. The instrument includes 19 items (eg, check whether information is current and updated recently), evaluating eHealth literacy from 3 dimensions.

Petrič et al [19] devised the expanded eHEALS-E based on the revised eHEALS, aiming to better cover the complicated factors contributing to eHealth knowledge popularization. This instrument is composed of 20 items, involving 6 dimensions. One example item is: “On the Internet, I prefer reading short and simple health explanations instead of complicated expert clarifications.”

Kayser et al [22] developed eHLQ on the foundation of the eHealth literacy framework, proposed by Norgaard et al [7], and meanwhile constructed this instrument in Denmark and English. This instrument is aimed at comprehensively measuring eHealth literacy from perspectives of individual knowledge and skill, digital service system operation, interaction between individual and digital system, etc. In total, 35 items (eg, technology improves my communication), totaling 7 dimensions, are incorporated in this instrument, which has been tested in Norway [47] and Serbia [48].

Paige et al [6] came up with the eHealth literacy model (Transactional Model of eHealth Literacy) in 2018, which is highlighting the transactional feature and focusing on the individual ability to communicate with others and exchange information while solving health problems. Based on this model, they established TeHLI [23] in 2019, which measures perceptual skills related to understanding, communication, appraisal, and application of web-based health information through 18 items in 4 dimensions. One example item is: I can use the Internet to learn about topics that are relevant to me.

Liu et al [25] held the view that, with the development of the internet, the Web 3.0 age puts more emphasis on the effective and organized application of digital new technology while integrating information. Therefore, the eHealth literacy assessment instrument, eHLS-Web3.0, was built under the background of Web 3.0, which has 3 dimensions and focuses on measuring the new requirements (namely, the capacity to apply mobile service) newly created by Web 3.0 with a total of 24 items (eg, when searching the health information on the internet, I will check who owns the website).

Yoon et al [26] developed DHTL-AQ by integrating existing assessment tools and complementing 10 common digital technology task abilities. This tool is designed to measure users’ ability to use various digital technologies such as computers, smartphones, mobile medical apps, wearable devices, and so on, in the clinical context. This instrument covers the 2 fields, digital functional and digital critical literacy, with 4 dimensions and 34 items (eg, I can record my health information through the app).

In addition, Rachmani et al [27] found that the existing eHealth literacy assessment instrument, eHEALS, did not involve the skill of interaction on the internet, and DHLI did not measure mobile health literacy skills. Hence, with the purpose of settling the measure dimension deficiency of the 2 instruments, they devised DHLC [27] on the basis of the Digital Competence Framework for Citizens (DigComp 2.1) [49] and digital health literacy [2], containing 26 items (eg, I can protect my social media [eg Twitter, Facebook, and Instagram] account such as using different methods [eg, a strong password and control the recent logins].), which are divided into 9 dimensions.

Overall, now there are many subjective assessment instruments, generally constructed according to the conceptual level of eHealth literacy. However, due to the discrepancy in the depiction of eHealth literacy connotation, the assessment instruments’ measuring dimension varies a lot. Additionally, subjective assessment instruments all adopt self-report measurement, and only the respondents’ views about their own eHealth literacy and capability are collected, which means the evaluation is not objective and may fail to appraise the level of eHealth literacy accurately.

#### Comprehensive Assessment Instruments

On account of the possible gap between the results obtained from subjective assessment instruments and the practical level, researchers attempted to combine self-report and direct assessment, adding the actual operation to evaluate eHealth literacy comprehensively. This study sorted out 4 comprehensive assessment instruments: RRSA [16], DHLI [20], eHLA [21], and DHLA [24]. The core skill assessment dimensions of each instrument are presented in Table 4.

**Table 4.** The core skill assessment dimensions of comprehensive assessment instruments.

Abbreviation of instruments and dimensions of assessment		Corresponding items, n
RRSA^a^ [16]		
	Finding health information	1 self-report item, 56 practical questions
	Evaluating health information	1 self-report item, 56 practical questions
	Understanding plagiarism	1 self-report item, 56 practical questions
DHLI^b^ [20]		
	Operational skills	3 self-report items and 1 task test item
	Navigation skills	3 self-report items and 1 task test item
	Information searching	3 self-report items and 1 task test item
	Evaluating reliability	3 self-report items and 1 task test item
	Determining relevance	3 self-report items and 1 task test item
	Adding self-generated content	3 self-report items and 1 task test item
	Protecting privacy	3 self-report items and 1 task test item
eHLA^c^ [21]		
	Functional health literacy	10
	Health literacy self-assessment	9
	Familiarity with health and health care	5
	Knowledge of health and disease	6
	Technology familiarity	6
	Technology confidence	4
	Incentives for engaging with technology	4
DHLA^d^ [24]		
	Self-assessment of digital health literacy	6
	How convincing people found internet health information from different sources	3
	Trust in health information from folklore and customs	1

a RRSA: Research Readiness Self-Assessment.

b DHLI: Digital Health Literacy Instrument.

c eHLA: eHealth Literacy Assessment.

d DHLA: digital health literacy assessment.

RRSA that was developed by Ivanitskaya et al [16] is divided into knowledge test and operation test, which examines respondents’ grasp of the basic knowledge of health information, retrieval capability, and the ability to correctly select network linking and authenticate information. This instrument includes 56 items that are objective questions with correct answers, containing 16 choice questions (eg, Which of the following titles are scholarly or academic journals?) and 40 judgment questions, and 1 self-assessment item evaluating that information retrieval ability is also encompassed. RRSA further measures participants’ practical skills in eHealth information perception and acquisition on the foundation of self-appraisal.

As digital technology advances, to expand the measuring range of eHealth literacy and encompass the necessary skills to use Health 1.0 and Health 2.0 tools, van der Vaart and Drossaert [20] developed DHLI in 2017. This instrument constructed in Dutch consists of 21 self-report items and 7 operational items (eg, Do you [intentionally or unintentionally] share your own private information [eg, name or address]?), covering 7 dimensions, and each dimension is composed of 3 self-report items and 1 operational item. The operational item inquires the respondent a question about internet operation skills. There is only 1 right answer in the options, and the final score is based on the number of correct answers.

Karnoe et al [21] invented the eHLA toolkit, in which there are 5 self-assessment tools and 2 objective assessment tools, measuring a total of 7 dimensions encompassing both health and digital aspects. Tool 1 and tool 4 are objective evaluation tools in the form of single-choice questions, scoring according to the number of correct answers. One example question is: Which of the following is one of the livers’ main functions? Tools 2, 3, 5, 6, and 7 are subjective evaluation tools designed to obtain respondents’ self-evaluation of related skills.

With the intention of filling the research gap in the risk of misinterpreting health information, Liu et al [24] developed DHLA on the basis of eHEALS in 2020 and constructed a web-based health information bank with correct and incorrect answers so as to appraise people’s risk of misinterpreting health information and accordingly dividing them into high-, medium-, and low-risk groups. In this instrument, there are 10 self-assessment items consisting of 3 dimensions and 5 judgment questions (2 simple ones, 2 medium ones, and 1 difficult one, eg, smokers can eat more pig blood, which will cleanse their lungs) randomly drawn from a web-based health information bank and given to participants to judge whether they are true or false.

Comprehensive assessment instruments are intended to reduce the bias caused by respondents overestimating or underestimating their eHealth literacy skills by introducing an objective assessment into self-report. Compared with subjective assessment instruments, the accuracy of evaluation results is improved. Nevertheless, due to the rapid development and change in the eHealth field, it is difficult to update standardized measurement items in the objective section constantly.

### Assessment Theme Analysis

To clarify the assessment range of the instruments, this review referred to the extraction methods of related assessment instrument themes [50] and conducted content analysis of the included 13 assessment instruments. The measured content of the 13 instruments was categorized into 21 themes: browsing, understanding, communication, search, acquisition, application, appraisal, writing, health awareness, familiarity with health, social support, attitude, initiative, self-efficacy, confidence, sharing, familiarity with technology, health management, netiquette, privacy security, and originality protection.

These themes can be further divided into 4 areas: skill factors, psychology factors, health factors, and information factors. The skills factors contain the 8 themes: browsing, understanding, communication, search, acquisition, application, appraisal, and writing; psychology factors encompass 5 themes: social support, attitude, initiative, self-efficacy, confidence, and sharing; health factors include 3 themes: health awareness, familiarity with health, and health management; and information factors comprise 4 themes: familiarity with technology, netiquette, privacy security, and originality protection. The themes involved in each instrument are presented in Table 5. The statistical results of the assessment instruments by theme are shown in Table 6.

Among the 13 eHealth literacy assessment instruments, DHLC [27] and eHLS-Web3.0 [25] measure the most themes, covering 13 and 12 themes, respectively; RRSA [16] is the least with only 4 themes measured. In total, 12 instruments were designed to test search ability and 12 instruments for appraisal ability, and acquisition and application are also frequently incorporated into assessment content. Besides, browsing, understanding, communication, writing, attitude, self-efficacy, familiarity with technology, health management, and privacy security are also tested to some extent, while health awareness and sharing only appear in 2 instruments; familiarity with health, social support, initiative, netiquette, and originality protection are mentioned only in 1 instrument. In the future, the development of assessment instruments can increase the measurement of the psychology factors and information factors.

**Table 5.** Assessment instruments’ themes distribution.

Themes		Study												
		Norman and Skinner [2]	Ivanitskaya et al [16]	Koopman et al [17]	Seçkin et al [18]	Petrič et al [19]	van der Vaart and Drossaert [20]	Karnoe et al [21]	Kayser et al [22]	Paige et al [23]	Liu et al [24]	Liu et al [25]	Yoon et al [26]	Rachmani et al [27]
Skill factors														
	Browsing				✓	✓		✓	✓				✓	✓
	Understanding					✓		✓	✓	✓				✓
	Communication				✓					✓		✓		✓
	Search	✓	✓	✓		✓	✓	✓	✓	✓	✓	✓	✓	✓
	Acquisition	✓	✓			✓	✓	✓	✓	✓	✓	✓	✓	✓
	Application	✓	✓				✓	✓	✓	✓	✓	✓	✓	✓
	Appraisal	✓	✓		✓	✓	✓	✓	✓	✓	✓	✓	✓	✓
	Writing						✓	✓	✓					✓
Psychology factors														
	Social support									✓				
	Attitude			✓	✓	✓		✓				✓		
	Initiative											✓		
	Self-efficacy	✓			✓	✓		✓	✓	✓	✓	✓		
	Confidence	✓			✓	✓		✓						
	Sharing											✓		✓
Health factors														
	Health awareness			✓					✓					
	Familiarity with health							✓						
	Health management									✓		✓	✓	✓
Information factors														
	Familiarity with technology			✓		✓	✓	✓				✓	✓	✓
	Netiquette													✓
	Privacy security			✓			✓		✓	✓			✓	✓
	Originality protection											✓		

**Table 6.** Theme statistics of assessment instruments.

Themes	Theme coverage count, n
Search	12
Appraisal	12
Acquisition	11
Acquisition	10
Self-efficacy	8
Familiarity with technology	7
Browsing	6
Privacy security	6
Understanding	5
Attitude	5
Confidence	5
Communication	4
Writing	4
Health management	4
Sharing	2
Health awareness	2
Social support	1
Initiative	1
Familiarity with health	1
Netiquette	1
Originality protect	1

## Discussion

### Principal Findings

Our study identified 13 eHealth literacy assessment instruments through literature search, collection, screening, and collation and conducted a scoping review. Since Norman and Skinner [28] put forward the concept of eHealth literacy in 2006, the development of eHealth literacy assessment instruments has undergone great advances. Although the first eHealth literacy assessment instrument eHEALS [2] focused on the knowledge related to health literacy in the digital environment, with the advance of information technology, the concept of eHealth literacy is in an evolving process. The skills needed to use eHealth services are expanding, and the concept thereupon reflected not only an individual’s one-way ability to use technology. Subsequent models and assessment instruments were refined to adapt to the evolving digital environment and to broaden the scope of eHealth literacy measurement. To achieve this, they incorporated evaluations of digital skills, such as familiarity with mobile apps, as well as digital abilities between individuals and technology and between individuals and eHealth services. These additions aimed to comprehensively address the new demands brought about by technological advancements.

The reliability coefficient results of the 13 eHealth literacy assessment instruments indicated their satisfactory performance on reliability, being able to provide relatively reliable and stable evaluation results. Whereas in the aspects of validity, because eHealth literacy is a multidimensional and multilevel concept, different design purposes and focus of the instruments resulted in different selections of validity test categories. Most assessment instruments tested content validity to ensure that the assessment content reflects all aspects of eHealth literacy accurately. Several instruments also dealt with structural validity to examine the rationality and stability of the instruments’ internal structure. Discriminant validity, convergent validity, and external validity were seldom involved in the assessment instrument examination.

Different eHealth literacy assessment instruments adopt self-report assessment or comprehensive assessment combined with self-report and actual operation. In comparison with comprehensive assessment, self-report is limited to examine respondents’ perceptive ability to their own eHealth literacy but unable to test their actual perception and technology use ability to eHealth programs. Partial studies [20,21] have recognized the importance of including objective evaluation criteria in eHealth literacy measurement to survey participants’ real ability level. Comprehensive assessment merges subjective and objective assessment, forming an assessment with diverse evaluation dimensions and abundant evaluation levels, which is beneficial to revealing respondents’ authentic level more precisely. However, how to reasonably allocate the weight of subjective evaluation and objective measurement is not mentioned in current studies.

### Implications for Future Studies on eHealth Literacy Instruments

#### Validation of Existing eHealth Literacy Assessment Instruments

As digital technology surges forward, the connotation of eHealth literacy has been constantly updated. Therefore, various eHealth literacy assessment tools emerged, and it is critical to use proper assessment instruments scientifically for accurate evaluation of eHealth literacy. A review based on the eHealth literacy assessment instrument of COSMIN [51] suggested that e-HLS [4], DHLI [6], eHLA [7], and eHLQ [1] have only been examined once or twice, lacking comprehensive evidence to demonstrate their performance. TeHLI [10] performed well in structural validity and internal consistency but has not been validated in other populations. Among the existing assessment instruments, apart from eHEALS [2], which has been translated into many languages and conducted reliability tests and used widely, other assessment instruments have not received examination and application in multipopulation and multicultural contexts. Hence, high-quality research is essential for conducting cross-linguistic and cross-population psychological measurements on existing assessment instruments. This will help expand the range of eHealth literacy tools suitable for different cultural backgrounds and populations.

#### Deepening and Integration of eHealth Literacy Connotation

Due to the discrepancies in the definitions of eHealth literacy among scholars, eHealth literacy assessment instruments developed on the basis of different theoretical models differ in content, making the measurement results incomparable. Moreover, various assessment instruments focus on a certain aspect of ability, failing to evaluate an individual’s eHealth literacy level comprehensively. Therefore, in the future, if current research on the connotation of eHealth literacy can be integrated with the relationships between individuals and information, technology, and environment, a more comprehensive and in-depth characterization of eHealth literacy can be achieved. This would help establish a unified definition, providing a solid foundation for developing assessment instruments and promoting their standardization.

#### Objectification of the eHealth Literacy Assessment Method

Current eHealth literacy assessment instruments mainly adopt self-reports of respondents to measure their eHealth literacy level, which reflects more on the individual’s cognition of self-eHealth literacy. However, such subjective assessment instruments are prone to eliciting response bias and overestimation of individuals’ perception of their eHealth literacy levels [52]. Research on the predictive validity of eHEALS [2] revealed that the correlation between an individual’s own perceived level and the actual performance on web-based health tasks is weak [29]. Hence, the future development of eHealth literacy assessment instruments should incorporate more objective test content. A reasonable number of items assessing concrete eHealth knowledge and skills should be added to create a comprehensive tool that integrates both subjective and objective components, combining self-assessment with external assessment. This approach would allow for a more objective and accurate evaluation of respondents’ eHealth literacy.

### Limitations

The main limitation of this review is that due to the limitations of the literature search strategy and database selection used, as well as the inclusion of only peer-reviewed journal papers published in English, the literature coverage might not be complete, which probably influenced the analysis results. Future work should focus on expanding current achievements.

### Conclusions

Assessment of eHealth literacy is the premise of researching and improving eHealth literacy. This study used a scoping review method to identify 13 eHealth literacy assessment instruments from the literature and analyzed them from the perspectives of the development process, instrument characteristics, and assessment theme.

Concerning the development process, there were only 2 assessment instruments in the incipient stage in 2006, and 11 more assessment instruments were devised after 2014. Different assessment instruments’ development underwent continuous adjustment both in content and form so as to reflect the constantly renewing eHealth literacy concept.

As for assessment dimension and score range, the 13 eHealth literacy assessment instruments contain a number of dimensions ranging from 3 to 9. The self-assessment sections are rated from 4 to 6, with a 5-level being the most common.

In the aspects of reliability and validity test of the assessment instruments, the 13 instruments all conducted reliability tests, and the results revealed that the assessment instruments performed well in consistency, stability, and dependability. Except for eHEALS [2], the other 12 instruments all reported evaluation validity. On account of the difference among the instruments in their depiction of eHealth literacy’s connotation and focus, the selected validity test category of the instruments varies. In total, 12 instruments involved content validity, structural validity, discriminant validity, external validity, and convergent validity to appraise assessment instruments’ effectiveness.

In terms of assessment method, subjective assessment instruments are the most with a total of 9 evaluating in the form of self-report, exemplified by the classic assessment instrument eHEALS [2], and it gained the widest application. There are only 4 comprehensive assessment instruments, which are devised on the basis of subjective assessment and incorporate actual operational test based on the scene in order to reflect individual-related capacity objectively.

The theme analysis of the 13 assessment instruments indicated that assessment instruments targeting search, evaluation, acquisition, and application ability account for the most. Browsing, understanding, communication, self-efficacy, familiarity with technology, and privacy security were also evaluated to a certain extent, while the psychology and information factors, such as sharing, social support, initiative, netiquette, originality protection, and so on, were less concerned.

The most widely used subjective assessment tool is eHEALS [2], which is more suitable for user self-reported empirical research. The comprehensive evaluation tool, such as DHLI [20], has multiple dimensions and rich levels of evaluation, which can meet diverse evaluation needs. Overall, subjective and comprehensive assessment tools for eHealth literacy have their own advantages and disadvantages. Subjective assessment tools have a friendly evaluation method, but their test validity is relatively low. There is a risk of time-consuming and low recognition for comprehensive evaluation tools. Therefore, it is necessary to scientifically select and use evaluation tools based on different research objectives or research plan designs.

## Supplementary material

10.2196/66965 Multimedia Appendix 1 Search strategy.

10.2196/66965 Checklist 1 PRISMA-ScR (Preferred Reporting Items for Systematic Reviews and Meta-Analyses Extension for Scoping Reviews) checklist.

## Abbreviations

DHLA: digital health literacy assessment
DHLC: digital health literacy competency
DHLI: Digital Health Literacy Instrument
DHTL-AQ: Digital Health Technology Literacy Assessment Questionnaire
e-HLS: Electronic Health Literacy Scale
eHEALS: eHealth Literacy Scale
eHEALS-E: Extended eHealth Literacy Scale
eHLA: eHealth Literacy Assessment
eHLQ: eHealth Literacy Questionnaire
eHLS-Web3.0: eHealth Literacy Scale-Web3.0
PRE-HIT: Patient Readiness to Engage in Health Internet Technology
PRISMA: Preferred Reporting Items for Systematic Reviews and Meta-Analyses
PRISMA-ScR: Preferred Reporting Items for Systematic Reviews and Meta-Analyses extension for Scoping Reviews
RRSA: Research Readiness Self-Assessment
TeHLI: Transactional eHealth Literacy Instrument
