# Supplementary material for: eHealth Literacy Assessment Instruments: Scoping Review
Source: J Med Internet Res. 2025 Aug 20;27:e66965. doi: 10.2196/66965 (PMC12367351; doi:10.2196/66965)
Supplement: Multimedia Appendix 1 [file jmir-v27-e66965-s001.docx]

Search Strategy Database: Web of Science (literature published Jan 1, 1900 and Jun 1, 2024)

| **#** | **Search Query** | **Results** |
| --- | --- | --- |
| **1** | TS=("ehealth literacy" OR "E-health literacy" OR "electronic health literacy") | 1,397 |
| **2** | TS=(Assessment* OR Measure* OR Tool* OR Test* OR Instrument* OR Questionnaire* OR Psychometric* OR Screen* OR Survey*) | 32,805,121 |
| **3** | #1 AND #2 | 979 |

Search Strategy Database: Scopus (literature published up to Jun 1, 2024)

| **#** | **Search Query** | **Results** |
| --- | --- | --- |
| **1** | ("ehealth literacy" OR "E-health literacy" OR "electronic health literacy") | 1,423 |
| **2** | (Assessment* OR Measure* OR Tool* OR Test* OR Instrument* OR Questionnaire* OR Psychometric* OR Screen* OR Survey*) | 30,383,799 |
| **3** | #1 AND #2 | 1,064 |

Search Strategy Database: PubMed (literature published Jan 1, 1900 and Jun 1, 2024)

| **#** | **Search Query** | **Results** |
| --- | --- | --- |
| **1** | ("ehealth literacy" OR "E-health literacy" OR "electronic health literacy") | 949 |
| **2** | (Assessment* OR Measure* OR Tool* OR Test* OR Instrument* OR Questionnaire* OR Psychometric* OR Screen* OR Survey*) | 11,897,184 |
| **3** | #1 AND #2 | 655 |

Search Strategy Database: EBSCO (literature published Jan 1, 1900 and Jun 1, 2024)

| **#** | **Search Query** | **Results** |
| --- | --- | --- |
| **1** | ("ehealth literacy" OR "E-health literacy" OR "electronic health literacy") | 3,244 |
| **2** | (Assessment* OR Measure* OR Tool* OR Test* OR Instrument* OR Questionnaire* OR Psychometric* OR Screen* OR Survey*) | 50,545,081 |
| **3** | #1 AND #2 | 274 |
